# Residual cancer burden in locally advanced breast cancer: a superior tool

**DOI:** 10.3747/co.v15i6.242

**Published:** 2008-12

**Authors:** Z. Nahleh, D. Sivasubramaniam, S. Dhaliwal, V. Sundarajan, R. Komrokji

**Affiliations:** * Wayne State University, Karmanos Cancer Institute, Detroit, MI, U.S.A; † University of Cincinnati, Cincinnati, OH, U.S.A

**Keywords:** Breast cancer, residual disease, pathology, endpoints, locally advanced disease

## Abstract

**Objectives:**

Locally advanced breast cancer (labc) poses a difficult clinical challenge with an overall poor long-term prognosis. The strength of the association between tumour characteristics, treatment response, and outcome is not well defined. In the present study, we attempted to gain further insight into labc by reviewing tumour characteristics of patients treated with neoadjuvant chemotherapy and by studying the association of those characteristics with outcome. We calculated the residual cancer burden (rcb) score obtained at surgery and attempted to study its correlation with event-free survival (efs) and overall survival (os).

**Methods:**

We studied patients diagnosed primarily with labc (*n* = 45). Pathologic and clinical responses were determined. Pathology slides were reviewed.

**Results:**

Of the 45 study patients, 9% had stage iib disease; 29%, stage iiia; 51%, stage iiib; and 11%, stage iiic. Inflammatory breast cancer (ibc) was found in 16%. Pathologic complete response (pcr) was achieved in 22% of all patients. None of the patients with ibc achieved pcr. Patients with estrogen receptor–negative (er−)/progesterone receptor–negative (pr−) tumours were more likely to achieve pcr than were those with er+/pr+ tumours. Among patients with tumours that overexpressed human epidermal growth factor receptor 2 (her2/*neu*), 17% achieved pcr as compared with 25% of patients with non-overexpressing tumours; only 1 patient had received trastuzumab. The rcb scores were calculated in 32 patients and ranged between 0 and 4.6.

**Conclusions:**

The present study examined practical issues related to the classification and management of labc and ibc. The rcb, defined from routine pathology materials, was easily quantifiable. It appears to be a better predictor than pcr of outcome following neoadjuvant chemotherapy in labc. Higher rcb scores were associated with lower efs and a lower rate of os. A continual quest for reliable predictive and correlative prognostic markers, and for better surrogate endpoints for outcome, is essential to advance our understanding of labc and to improve treatment outcomes.

## 1. INTRODUCTION

Since the late 1970s, tremendous progress has been achieved in the understanding and management of breast cancer. However, locally advanced breast cancer (labc) remains a difficult clinical challenge, with a long-term survival rate of less than 50%^1^. Treatment of labc uses a multimodality approach involving chemotherapy, surgery, and radiation therapy 2. The optimal neoadjuvant chemotherapeutic regimen for labc continues to evolve. The aim of neoadjuvant chemotherapy is to decrease tumour bulk and ideally to achieve complete clinical and pathologic responses.

Pathologic complete response (pcr) has been viewed as a reliable primary endpoint for outcome and survival following neoadjuvant chemotherapy for breast cancer 3,4. However, residual disease may encompass a range of pathologic responses likely encompassing a variety of prognostic groups from near-complete response to resistance. Therefore, additional surrogate endpoints for outcome and survival are needed. Also, many of the classic biologic predictive and prognostic factors such as hormone receptors and tumour grade may have implications that are different in labc than in earlier stages of the disease 5–9. To advance our understanding of this disease, identification of reliable markers that would lead to better disease classification and improved treatment outcomes is desirable. However, few trials studying primary (preoperative) chemotherapy have focused exclusively on patients with locally advanced disease.

In the present observational study, we attempted to gain further insight into labc by reviewing tumour characteristics in patients treated with neoadjuvant chemotherapy at a single institution, and by studying the association of those tumour characteristics with outcome. We were specifically interested in determining the practicality of calculating the residual cancer burden (rcb) scores obtained at surgery and in studying the correlation of those scores with event-free survival (efs) and overall survival (os) as compared with pcr.

The rcb index was proposed by Symmans *et al.* 10 as a determinant of the extent of residual disease in the post-treatment surgical resection specimen of patients with breast cancer who received preoperative chemotherapy. The rcb index was found to be an improvement over currently used risk factors for the prediction of distant relapse after neoadjuvant chemotherapy. If independently validated, the rcb index is suggested to provide an accurate surrogate endpoint for patient survival.

The rcb index is determined from

- the bi-dimensional diameters of the primary tumour bed in the resection specimen (*d*_1_ and *d*_2_),
- the proportion of the primary tumour bed that contains invasive carcinoma (*f*_in_),
- the number of axillary lymph nodes containing metastatic carcinoma (*LN*), and
- the diameter of the largest metastasis in an axillary lymph node (*d*_met_).

Largest bi-dimensional measurements of the residual primary tumour bed are recorded from the macroscopic description and are combined as follows:

[1] $$d_{\text{prime}}={\left(d_{1}d_{2}\right)}^{1/2}.$$

The proportion of invasive carcinoma (*f*_inv_) within the cross-sectional area of the primary tumour bed is estimated from the overall percentage area of carcinoma (%CA) and is then corrected for the component of *in situ* carcinoma (%CIS):

[2] $$f_{\text{inv}}=[1-(\%\text{CIS}/100)]\times (\%\text{CA}/100).$$

Symmans *et al*. calculated rcb indexes based on a review of patients who completed neoadjuvant chemotherapy for invasive breast carcinoma (T1–3, N0–1, M0) at the M.D. Anderson Cancer Center 10. We reviewed pathology slides and reports from 432 patients in two completed neoadjuvant trials:

- Fluorouracil, doxorubicin, and cyclophosphamide (fac) in 189 patients
- Paclitaxel followed by fac (t/fac) in 243 patients.

The rcb was calculated as an index that combines pathology measurements of the primary tumour (size and cellularity) and nodal metastases (number and size). Four rcb categories [rcb-0 (pcr) to rcb-3 (chemoresistant)] and post-treatment revised American Joint Committee on Cancer (ajcc) stage (0–iii) for prediction of distant relapse-free survival (drfs) were compared in multivariate Cox regression analyses stratified by estrogen receptor status (er) status.

The RCB was found to be a continuous predictor of drfs and to predict relapse more strongly than ajcc stage did. In univariate Cox regression analyses, the four parameters of residual tumour (*d*_prim_, *f*_inv_, LN, and *d*_met_) were individually associated with significantly higher risk of distant relapse (*p* < 0.001) after t/fac chemotherapy. They maintained significance as independent predictors in the main-effects multivariate Cox regression model. Patients had an almost-doubled relapse risk for each unit of increase in the rcb index [hazard ratio (hr): 1.94; 95% confidence interval (ci): 1.47 to 2.55; *p* < 0.001]. When the rcb index was included in a multivariate Cox regression model that included clinical and treatment covariates, the overall predictive power of the model was significantly improved (*p* < 0.001), and the rcb index was significantly associated with the risk of disease recurrence (hr: 2.50; 95% ci: 1.70 to 3.69; *p* < 0.001).

## 2. PATIENTS AND METHODS

### 2.1 Selection Procedures

After obtaining approval from the University of Cincinnati institutional review board, we conducted a retrospective chart review of the breast oncology database and reviewed the medical records of patients who received neoadjuvant chemotherapy at the University of Cincinnati between January 1, 2001, and December 31, 2005. We included consecutive patients diagnosed primarily with inoperable labc staged as iib, iiia (T0–N2; T1/2–N2; T3–N1/2), iiib (T4, N0–2), or iiic disease (any T, N3). Patients with inflammatory breast cancer (ibc) were included. We excluded patients diagnosed with operable tumours staged as i, iia, and iib, even if they received neoadjuvant chemotherapy. Patients with stage iv disease were also excluded.

Initially, we identified 50 patients; 5 were later excluded when found to have metastatic disease on staging workup. We evaluated 45 patients. Tumour and patient characteristics were reviewed (Table I). Patients were divided into 4 treatment groups based on their neoadjuvant chemotherapy regimens:

- Anthracycline (doxorubicin or epirubicin) plus taxane (paclitaxel or docetaxel)
- Anthracycline only
- Single-agent taxane
- Other regimens [cyclophosphamide, methotrexate, and 5-fluorouracil (cmf), capecitabine, and so on]

Treatment with trastuzumab was also noted.

### 2.2 Tumour Response

Clinical response was recorded before each chemotherapy cycle and before surgery. No clinical evidence of palpable tumour in the breast and axillary lymph nodes was defined as a clinical complete response (ccr), reduction in total tumour size of 50% or more was graded as a clinical partial response (cpr). An increase in total tumour size of more than 50% or the appearance of new suspicious ipsilateral axillary adenopathy was considered progressive disease. Tumours that did not meet the criteria for objective response or progression were considered stable disease.

Pathologic response was determined at surgery. A pcr was defined as no invasive tumour in breast or axillary lymph nodes. Complete response in breast, but residual disease in lymph nodes was designated rdln; residual disease in breast, but no disease in lymph nodes was designated rdb; and residual disease in both was designated rdbln.

### 2.3 Calculation of Residual Cancer Burden

Pathology slides for 32 of the 45 patients were available. The characteristics of these 32 patients were very similar to those of the whole group (Table II). The slides were retrieved, reviewed, and analyzed by our pathologist (VS) for various parameters that are required to calculate rcb 10, including

- the largest two dimensions (in millimetres) of the residual tumour bed in the breast (largest tumour bed if multicentric disease).
- histologic mapping of the entire largest cross-sectional area of the residual tumour bed, with specific identification of the relevant slides in the pathology report.
- histologic assessment of the percentage of the tumour bed area that contains carcinoma (all carcinoma—that is, invasive and *in situ*), selected as one of 0%, 1%, 5%, 10%, 20%, 30%, 40%, 50%, 60%, 70%, 80%, or 90%.
- histologic estimate of the percentage of the carcinoma in the tumour bed that is *in situ*, selected as one of 0%, 1%, 5%, 10%, 20%, 30%, 40%, 50%, 60%, 70%, 80%, or 90%.
- number of positive (metastatic) lymph nodes.
- largest diameter (in millimetres) of the largest nodal metastasis.

These variables were entered into the M.D. Anderson Residual Cancer Burden Calculator (found online at www3.mdanderson.org/app/medcalc/index.cfm?pagename=jsconvert3).

### 2.4 Statistics

Data were entered and analyzed using SPSS biostatistical software package (SPSS, Chicago, IL, U.S.A.), version 10.0.05. The study aimed primarily to evaluate the feasibility of rcb calculation from standard pathology specimens. It also aimed to study the association of rcb and pcr with efs, defined as time to breast cancer recurrence, itself defined as local lymph node or breast recurrence, metastasis to other sites, second primary breast cancer, or any death. Analyses of os were also performed; os included all deaths whether they were breast cancer–related or not. Patient and disease characteristics between the different groups were compared using simple log-rank tests and Cox proportional hazards models. The efs was considered in a multivariable setting with Cox proportional hazards models. Race, hormone receptor status [er+/progesterone receptor positive (pr+), er+/pr−, er−/pr+, or er−/pr−], chemotherapy (anthracycline, anthracycline and taxane, taxane, others, and trastuzumab), stage (iib, iiia, iiib, iiic, and inflammatory), and human epidermal growth factor receptor 2 (her2/*neu*) were included in the multivariable model. The os and efs were estimated using the Kaplan–Meier product-limit method. The two-sided log-rank test was used to compare survival between pcr and rcb.

## 3. RESULTS

### 3.1 Patient Characteristics and Treatment

The median age of the 45 patients in the study was 51 years; 40% (*n* = 18) were white, and 60% (*n* = 27) were black. Stages were distributed as follows: 9% stage iib, 29% stage iiia, 51% stage iiib, and 11% stage iiic. Tumour types were 75% invasive ductal, 9% invasive lobular, and 16% ibc. In 47% of patients, tumours were er+ or pr+, distributed as follows: 18% er+/pr+, 27% er+/pr−, 2% er−/pr+, and 53% er−/pr−. Tumours positive for her2/*neu,* defined as immunohistochemical staining of 3+ or a fluorescent in-situ hybridization ratio above 2.2 for the her2/*neu* gene to chromosome 17, were identified in 27% of patients. Neoadjuvant chemotherapy regimens included doxorubicin or epirubicin plus taxane (paclitaxel or docetaxel), 80%; anthracycline-only, 10%; single-agent taxane, 4%; and other regimens (2 cmf, 1 capecitabine), 6%. One patient with her2/*neu-*positive disease received trastuzumab in combination with chemotherapy.

### 3.2 Principal Outcomes

**FIGURE 1:**
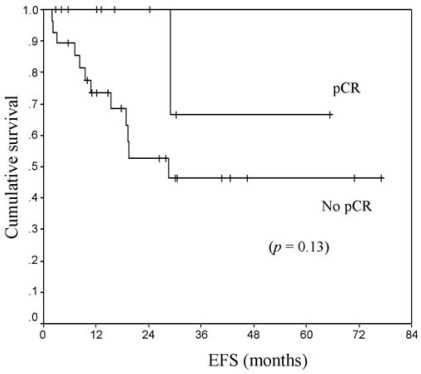
Event-free survival (efs) by pathologic complete response (pcr vs. No pcr) to neoadjuvant chemotherapy in locally advanced breast cancer.

**FIGURE 2:**
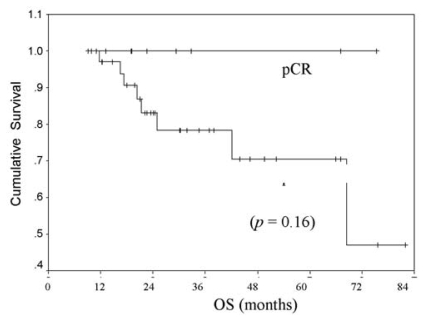
Overall survival (os) by pathologic complete response (pcr vs. no pcr) to neoadjuvant chemotherapy in locally advanced breast cancer.

**FIGURE 3:**
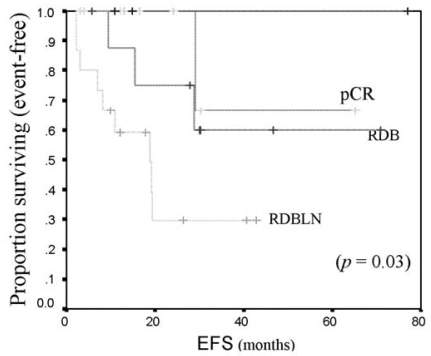
Event-free survival (efs) by pathologic complete response (pcr) to neoadjuvant chemotherapy in locally advanced breast cancer. rdb = residual disease in breast; rdbln = residual disease in breast and lymph nodes.

Table III shows responses to neoadjuvant chemotherapy. Clinical responses were distributed as follows: 55% (*n* = 25) achieved ccr; 38%, cpr; 4%, stable disease; and 2%, progressive disease. Pathologically, pcr was achieved in 22% (*n* = 10) of all patients. Among those patients, 7% had rdln; 24%, rdb; and 47%, rdbln. None of the patients with ibc achieved pcr. Among patients with er+ or pr+ tumours, 19% achieved pcr, as compared with 25% of patients with er−/pr− tumours. Among patients with her2/*neu-*positive tumours, 17% achieved pcr as compared with 25% patients whose tumours were her2/*neu-*negative. Among all patients who achieved ccr, only 36% achieved pcr. In patients who achieved pcr, os and efs were not yet reached at the study duration, as compared with 5.7 years and 19 months respectively for patients who did not achieve pcr (Figures 1 and 2). Patients who achieved pcr had the best efs; however, patients with residual disease in breast and lymph nodes appeared to have the worst outcomes (Figure 3).

Pathology slides for 32 patients were available for examination and calculation of rcb. The characteristics of these patients were comparable to those of the study population (Table II). The resulting rcb indexes ranged between 0 and 4.6. In 22% of patients (*n* = 7) the rcb index was 0; in 19% (*n* = 6), it was 1 < rcb < 2; in 10% (*n* = 3), it was 2 < rcb < 3; in 25% (*n* = 8), it was 3 < rcb < 4; and in 25%(*n* = 8), it was >4. In univariate Cox regression analysis, rcb correlated with efs (hr: 1.57; 95% ci: 1.04 to 2.38; *p* = 0.018) and with os (hr: 1.74; 95% ci: 0.91 to 3.32; *p* = 0.09). On the other hand, pcr did not seem to correlate with either efs (hr: 0.24; 95% ci: 1.86 to 2.38; *p* = 0.172) or os (hr: 0.03; 95% ci: 0 to 89; *p* = 0.40). In multivariate Cox regression analysis, rcb was noted to be an independent predictive variable for efs (hr: 1.59; 95% ci: 1.04 to 2.43; *p* = 0.033), but pcr was not (hr: 0.90; 95% ci: 0.52 to 1.57; *p* = 0.7).

## 4. DISCUSSION

Our study highlights a few practical points pertaining to the management of labc, including the overall treatment outcome in labc, the need for a better classification of labc, and the potential advantage of the rcb index as a better endpoint to measure response.

With regard to treatment outcome, labc continues to pose a significant clinical challenge, with standard available chemotherapy resulting in clinical and pathologic crs in only a very few patients. In our study, clinical response following neoadjuvant chemotherapy did not well predict pathologic response. Of all patients, 22% achieved pcr, a result that is essentially consistent with other trials in labc (mostly using standard anthracycline–taxane combination chemotherapy) 11–14. None of the patients in the present study with ibc achieved pcr, indicating the aggressive—and probably distinct—nature of this disease entity that begs for novel treatment strategies.

The optimal treatment algorithm, schedule, and mode of drug delivery in labc needs to be determined. The best outcome yet reported in a randomized phase iii trial in this patient population was obtained with metronomic chemotherapy given in protracted low doses 14 as reported by the Southwest Oncology Group. More research is needed to optimize treatment strategies so as to improve outcomes in labc and ibc.

A better classification of labc is also of paramount significance. The general term labc includes stage iiia (T0 N2; T1/2 N2; T3 N1/2), stage iiib (T4, N0–2), and stage iiic (any T, N3) tumours. Some of the classical biologic prognostic factors such as size and lymph node invasion have implications that are similar in labc to their implication in earlier disease stages. However, many others differ in labc. For example, the prognostic significance for hormone receptor status and her2/*neu* is unclear. In the present study, among patients with hormone-responsive tumours, only 19% achieved pcr as compared with 25% for patients with er−/pr−tumours, indicating favourable chemosensitivity of hormone non-responsive tumours to neoadjuvant chemotherapy in labc. In an evaluation of 124 patients with stage iii breast cancer, Stewart and others 5 found that, among patients with inoperable tumours, er status had no effect on prognosis. Other studies suggested that er− tumours are more chemosensitive than er + tumours are 11–15. In the present study, among patients with her2/*neu-*positive tumours, 17% achieved pcr as compared with 25% whose tumours were her2/*neu-*negative.

The patients included in our study were treated before the use of trastuzumab became routine in the neoadjuvant setting. The foregoing result will therefore likely improve with that change in clinical practice, as did outcomes reported in operable her2/*neu-*positive breast cancer with the addition of trastuzumab 16. Overexpression of her2/*neu*, otherwise known to be a poor prognostic factor, was found to be a predictor of a higher pcr with trastuzumab-based treatment 16 It is therefore evident that reliable predictive and correlative prognostic markers for outcome are essential to the individualization and improvement of treatment outcomes in labc.

The search for such markers is by no means simplistic. It likely requires application of optimal molecular classification methods; different combinations of the various predictors are likely to lead to different prognostic entities with different treatment outcomes. Investigators at M.D. Anderson 6 and the University of Carolina 7 independently examined chemosensitivity in basal-like breast cancers, which are also known by the clinical proxy “triple negative” (er−, pr−, her2/*neu-*negative) 8 Clinical response to neoadjuvant doxorubicin and cyclophosphamide was significantly higher among basal-like (86%) than among non-basal-like (her2 68%, luminal 60%) breast cancers. Similarly, pcr occurred in 30% of basal-like, 27% of her2/*neu-*positive, and 13% of luminal breast cancers 7. However, basal-like breast cancers have a poor prognosis, which seems paradoxical given their sensitivity to chemotherapy. The difference in outcome appears to be a result of the more frequent early relapses seen among basal-like and her2/*neu-*positive tumours that fail to achieve pcr. On the other hand, poor prognosis reflects the fewer treatment options available for er−, pr−, and her2/*neu-*negative tumours and the intrinsic biology of this subtype, which exhibits a high rate of relapse if complete eradication is not achieved and a poor outcome once relapse occurs 7. That understanding suggests that the basal-like and her2/*neu* subtypes that make up the preponderance of er− tumours are the tumours most affected by improvements in chemotherapy.

Other prognostic markers that may have different implications in labc include tumour nuclear grade, with poorly differentiated tumours being more likely than well differentiated tumours to respond to neoadjuvant chemotherapy 9,17. Also, increased expression of the human nuclear protein Ki-67, which is associated with cell proliferation and is used in routine pathology as a “proliferation marker” to measure the growth fraction of cells in human tumours, has been correlated with a better response to chemotherapy 18,19.

The main purpose of our study was to determine the ease and practical application of the rcb index in clinical practice as a more comprehensive and informative endpoint for residual disease following preoperative chemotherapy, based on data reported by Symmans and his colleagues at M.D. Anderson Cancer Center 10. The identification of reliable treatment endpoints is of crucial importance. It seems logical that a good response to chemotherapy in labc would predict for better long-term prognosis and ultimately for survival. The achievement of a pcr has been viewed as an acceptable primary endpoint for outcome following neoadjuvant chemotherapy for labc; patients who achieve a pcr to neoadjuvant chemotherapy for breast cancer have an improved prognosis 4,20–23.

In our study, the achievement of a pcr was associated with better os and efs. The prognostic significance of a pcr has been confirmed recently in a large published experience including 1731 patients from M.D. Anderson 3. However, a large trial involving 2411 patients with operable breast cancer did not show that improving the pcr affects os significantly. The adoption of os as a primary endpoint probably limited the ability of that trial to demonstrate a survival benefit, and os may not have been an ideal endpoint because the trial was not powered to detect such small differences in os or in disease-free survival^15^.

Despite the focus on pcr as a surrogate endpoint in neoadjuvant trials, logic seems to suggest that non-pcr patients may derive clinical benefit from regression of the primary tumour, even if survival is not proved to be affected. Symmans *et al.* studied the concept of residual cancer burden (rcb) in a study including 432 patients with operable breast cancer who completed neoadjuvant chemotherapy at M.D. Anderson 10. The rcb index was a continuous predictor of drfs, and it predicted relapse more strongly than ajcc stage did.

Our study found that rcb, defined from routine pathology materials, was easily quantifiable and appears to be better than pcr at predicting outcome after neoadjuvant chemotherapy in labc. Higher rcb scores were significantly associated with lower efs and a trend toward a lower rate of os. The rcb index maintained significance as an independent predictor of efs in the main-effects multivariate Cox regression model. What is interesting is that, in multivariate analysis, pcr did not maintain its significance as an independent predictor of efs. That result would suggest that more meaningful prognostic implications could be derived from rcb scores in patients undergoing neoadjuvant therapy.

Neither the rcb nor the pcr was statistically associated with os—an expected result, because of the small number of patients and limited number of events in the short duration of study between 2001 and 2005, which precludes accurate os assessment.

Prospective trials are needed to further evaluate the role of rcb as an endpoint following primary chemotherapy for labc. Because of the small number of patients and limited number of events in each group, it is not possible to draw definitive conclusions from the present study. Further analyses of other databases are required to confirm our finding of no difference in disease-free and overall survival between patients with residual ductal carcinoma *in situ* and those with no invasive or *in situ* disease following neoadjuvant chemotherapy for breast cancer.

## 5. CONCLUSIONS

A better stratification of labc based on specific markers is needed. The search for reliable predictive and correlative prognostic markers for outcome is essential to advance our understanding of this disease entity and consequently to improve treatment outcomes. However, the identification of reliable, informative, uniform endpoints is an essential first step that would also strengthen confidence in the value of neoadjuvant trials and anticipate the results of larger adjuvant trials. The classification of residual disease based on various pathologic responses may better classify the prognostic groups and would help to improve and individualize targeted treatment strategies. The rcb index, an easily quantifiable system, has the potential of providing a uniform method for reporting pathologic response with broad applicability.

## Figures and Tables

**Table I:** Patient characteristics

Characteristic	% (n)
Race	
White	40 (18)
Black	60 (27)
Stage	
iib	9 (4)
iiia	29 (13)
iiib	51 (23)
iiic	11 (5)
Histology	
Ductal	75 (34)
Lobular	9 (4)
Inflammatory	16 (7)
Hormone receptor	
er+/pr+	18 (8)
er+/pr−	27 (12)
er−/pr+	2 (1)
er−/pr−	53 (24)
her2/*neu*	
Positive	27 (12)
Negative	73 (33)

er = estrogen receptor; pr = progesterone receptor; her2/*neu* = human epidermal growth factor 2.

**Table II:** Patient characteristics of the study patients and of the whole group

Characteristic	Study patients	All patients
Patients (*n*)	32	45
Race (%)		
White	38	40
Black	62	60
Stage (%)		
iib	12	9
iiia	19	29
iiib	53	51
iiic	16	11
Histology (%)		
Ductal	72	75
Lobular	6	9
Inflammatory	22	16
Hormone receptor (%)		
er+/pr+	19	18
er+/pr−	25	27
er−/pr+	3	2
er−/pr−	53	53
her2/*neu* (%)		
Positive	28	27
Negative	72	73

er = estrogen receptor; pr = progesterone receptor; her2/*neu* = human epidermal growth factor 2.

**Table III:** Clinical and pathologic response after neoadjuvant chemotherapy

Response	% (n)
Clinical	
Complete response	55 (25)
Partial response	39 (17)
Stable disease	4 (2)
Progressive disease	2 (1)
Pathologic	
Complete response	22 (10)
Residual disease in lymph nodes	7(3)
Residual disease in breast	24 (11)
Residual disease in nodes and breast	47 (21)
